# Cherry-picking the Wrong Patients?

**DOI:** 10.1007/s00062-020-00878-2

**Published:** 2020-02-06

**Authors:** Jens Fiehler, Götz Thomalla, Martin Bendszus

**Affiliations:** 1 Department of Neuroradiology, University Medical Center Hamburg-Eppendorf, Hamburg, Germany; 2 Department of Neurology, University Medical Center Hamburg-Eppendorf, Hamburg, Germany; 3 Department of Neuroradiology, Heidelberg University Hospital, Heidelberg, Germany

Currently, several randomized controlled trials (RCTs) are enrolling patients with a focus on patients with low Alberta Stroke Program Early CT Score values (ASPECTS), such as TENSION (NCT03094715), TESLA (NCT03805308), and IN EXTREMIS [1, 2]. If theses RCTs confirm a thrombectomy treatment effect in patients with large infarcts, the imaging requirements for patient selection would decrease leading to speeding up the treatment processes and outcomes for all patients and further enlarge the overall number of patients eligible for thrombectomy.

In their letter the Calgary stroke team reported results of their recent survey on the influence of age on endovascular treatment decision-making and the willingness to randomize patients with low ASPECTS [3]. This survey was conducted during an interventional course, with the vast majority of participants being interventionalists. The results clearly showed a considerable reluctance among interventionalists to withhold thrombectomy treatment in younger patients suggesting that younger patients are much more likely to be treated outside the RCT and thus would be underrepresented in RCTs. This opinion is quite alarming as it would harm the generalizability of RCTs by introducing a selection bias generating a potential treatment effect size decrease and a false negative study result.

**Fig. 1:**
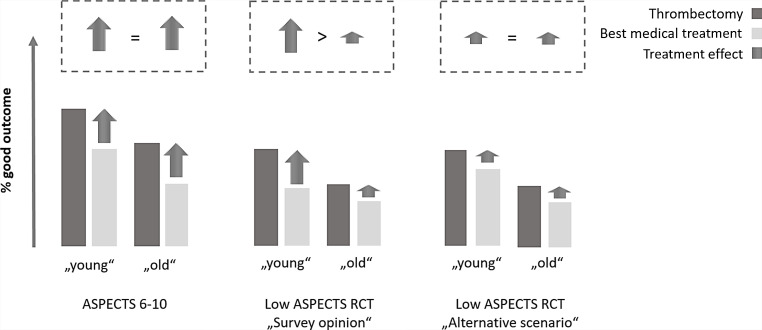
Hypothetical scenarios of trial outcomes, all of which are compatible with the observations of poorer outcomes in older patients. The only difference between “survey opinion” (middle) and “alternative scenario” is the rate of good outcome in young patients treated with best medical treatment alone

Undoubtedly, higher age decreases the chances of a good clinical outcome, particularly in patients with a low ASPECTS [4, 5]. It is not established, however, that the thrombectomy treatment effect decreases with increasing age [6]. Actually, the HERMES results showed no indications of a lower treatment effect in older patients [4]. It is important to avoid confusing the rate of good outcome, known from clinical experience, and the actual treatment effect versus best medical treatment alone (Fig. 1). Younger patients have a better outcome but the outcome is also better when treated with best medical treatment alone.

The clinical experiences of better clinical outcomes in younger than in older patients are perfectly compatible with higher treatment effects in the younger patient group. This hypothesis is reflected implicitly by the survey opinion (Fig. 1); however, the clinical observations are equally compatible with the same effect size in older and younger patients (Fig. 1). Even if the “survey opinion” is more intuitive, the alternative scenario is realistic as well.

Besides the individual responsibility of the physician of correctly selecting patients for the study, all studies should require a logfile in which all eligible patients (e.g. ASPECTS 3‑5) are documented, independent of the fact if they were included in the trial or not (as it is the case in TENSION). Thereby, a potential selection bias can be identified during the study with the chance to notify respective centers. Also, in retrospect an effect of cherry-picking on the results of the study can be identified.

The alarming results of the survey should be considered with some caution. First, the results are based on an informal voting during a meeting of interventionalists, which might not reflect the opinion of all neuroradiologists. Furthermore, this survey was exclusively done with interventional neuroradiologists. Stroke neurologists, who frequently enroll patients in trials may have voted differently and were not included.

Physicians involved in patient enrolment have a high responsibility and should avoid cherry-picking because of its profoundly negative impact on the external validity of the trial results. The study results will impact tens of thousands of stroke patients in the years to come. Moreover, cherry-picking of patients rests on a weak scientific foundation. Actually, it is not known who the “cherry patients” actually are, yet.
