# Correction to: Cherry-picking the Wrong Patients?

**DOI:** 10.1007/s00062-021-01028-y

**Published:** 2021-06-21

**Authors:** Jens Fiehler, Götz Thomalla, Martin Bendszus

**Affiliations:** 1 Department of Neuroradiology, University Medical Center, Hamburg-Eppendorf, Hamburg, Germany; 2 Department of Neurology, University Medical Center, Hamburg-Eppendorf, Hamburg, Germany; 3 Department of Neuroradiology, Heidelberg University Hospital, Heidelberg, Germany

**Correction to:**

**Clin Neuroradiol 2020**

10.1007/s00062-020-00878-2

The article “Cherry-picking the Wrong Patients?” by Jens Fiehler, Götz Thomalla, and Martin Bendszus, was originally published electronically on the publisher’s internet portal on 6 February 2020 without open access. With the author(s)’ decision to opt for open choice the copyright of the article changed on 21 April 2021 to “© The author(s) 2020” and the article is forthwith distributed under a Creative Commons Attribution 4.0 International License, which permits use, sharing, adaptation, distribution and reproduction in any medium or format, as long as appropriate credit is given to the original author(s) and the source, provide a link to the Creative Commons licence, and indicate if changes were made.

